# To BrAIST or not to BrAIST: decisions and characteristics of 1131 patients eligible for the Bracing in Adolescent Idiopathic Scoliosis Trial

**DOI:** 10.1186/1748-7161-7-S1-O23

**Published:** 2012-01-27

**Authors:** L Dolan, S Weinstein

**Affiliations:** 1 University of Iowa, Department of Orthopaedic Surgery, Iowa City, USA

## Background

BrAIST is a partially-randomized trial comparing the outcomes of bracing and observation in children with adolescent idiopathic scoliosis. The purpose of this study is to evaluate 1) whether the BrAIST sample is representative of the target population and 2) whether the treatment arms are equivalent. We addressed these questions by comparing baseline demographic, radiographic and psychosocial characteristics between the groups.

## Materials and methods

Since April 2007, 1131 patients met eligibility criteria; 360 (32%) agreed to participate. There were no statistically significant differences between those who declined and those who agreed to participate in terms of largest Cobb angle, curve type, gender, or age. Blacks/African-Americans were more likely to participate (50%) than other racial groups (p<0.01).

## Results

Of the 360, 219 (61%) entered into the bracing arm. Before treatment, there were no statistically significant differences in demographics, curve characteristics (Cobb angle, curve type, rotation, kyphosis, lordosis), skeletal maturity, general health, back pain and psychosocial characteristics including body image and quality of life. However, those who were very dissatisfied with their current back condition were more likely to choose a brace (73 vs. 51%, p<0.01).

## Conclusions

BrAIST is still open to enrollment and these results are preliminary. At this point, the sample appears representative of the target population of high-risk adolescents, indicating BrAIST results can be generalized outside this sample. Without complete randomization, the equivalence of the two treatment arms is therefore not guaranteed, but the fact that we found no significant differences in this analysis provides some confidence for minimal selection bias in the final results.

